# Post-pancreatectomy chemotherapy improves the survival of patients with late-stage but not early-stage pancreatic cancer

**DOI:** 10.3389/fonc.2026.1744027

**Published:** 2026-04-13

**Authors:** Lin Zhang, Feng Xue, Yali Cheng, Meirong Hou, Shuai Wu, Jiaqiang Ren, Zhenhua Ma, Zheng Wang, Rongqian Wu, Zheng Wu

**Affiliations:** 1 Department of Hepatobiliary Surgery, First Affiliated Hospital of Xi’an Jiaotong University, Xi’an, Shaanxi, China; 2 National Local Joint Engineering Research Center for Precision Surgery and Regenerative Medicine, Shaanxi Provincial Center for Regenerative Medicine and Surgical Engineering, First Affiliated Hospital of Xi’an Jiaotong University, Xi’an, Shaanxi, China

**Keywords:** adjuvant chemotherapy, early stage, pancreatectomy, pancreatic cancer, survival

## Abstract

**Background:**

Although current clinical guidelines recommend that patients who have undergone surgical resection for pancreatic cancer consider adjuvant chemotherapy options, a significant proportion of pancreatic cancer patients undergo surgical resection without receiving adjuvant chemotherapy. One key factor that contributes to this phenomenon is the uncertain effectiveness of adjuvant chemotherapy across various disease stages. The objective of this study was to explore the influence of postoperative chemotherapy on the prognosis of patients with different stages of pancreatic cancer.

**Methods:**

We retrospectively analyzed the clinical data of 405 pancreatic cancer patients who underwent pancreatectomy between February 2016 and December 2020 in First Affiliated Hospital of Xi’an Jiaotong University. After excluding patients who did not undergo surgery, received other treatments, or died within 30 days postoperatively, 258 patients were included.96 received adjuvant chemotherapy and 162 did not. Early-stage (stage I, n=59) and late-stage (stages II-IV, n=199) were based on AJCC 8th edition. To minimize intergroup differences, propensity score matching (PSM) was performed. Overall survival (OS) was analyzed using the Kaplan-Meier method and Cox regression analysis.

**Results:**

Among the 258 patients, 187 died during follow-up, and the median survival was 14.9 months. Median follow-up was 36.5 months. In the overall cohort, patients receiving chemotherapy had longer median OS than those who did not (17.2 vs 13.3 months; P < 0.001). Chemotherapy significantly improved median survival in late-stage disease (15.6 vs. 11.9 months, P = 0.03), but not in stage I disease (28.0 vs. 27.0 months, P = 0.45). After propensity score matching, multivariable analysis confirmed that chemotherapy was independently associated with improved OS in late-stage disease (HR 0.59; 95% CI 0.38–0.91; P = 0.02). However, no significant survival benefit was observed in stage I patients (HR 0.69; 95% CI 0.27–1.77; P = 0.45).

**Conclusions:**

Patients with late-stage pancreatic cancer benefit from adjuvant chemotherapy after pancreatectomy. However, in patients with stage I pancreatic cancer, our data suggest that adjuvant chemotherapy may not confer a significant survival benefit.

## 1 Introduction

Pancreatic cancer is an important cause of cancer-related death (1, 2). Current strategies for treating pancreatic cancer include neoadjuvant chemotherapy, surgery, and adjuvant radiation/chemotherapy (3–5). Among these treatments, surgery is considered the only possible curative option. Depending on the extent of local blood vessel involvement in primary tumors, pancreatic cancer can be categorized along a continuum from “unresectable” to “resectable” (6). Recent advances in surgical technology have made it possible for pancreatic cancer to be safely resected at many medical centers. However, the 5-year survival rate of postoperative patients who undergo R0 resection is only 10–25% (7). As such, the identification of effective postoperative therapy has become increasingly important (8–11).

Adjuvant chemotherapy is one of the most important measures for prolonging the survival of patients with pancreatic cancer after resection (12). Nevertheless, it remains uncertain what potential benefits adjuvant chemotherapy may offer to patients with different stages of pancreatic cancer. Early-stage pancreatic cancer is defined as stage I, and late-stage PDAC refer to pancreatic cancer of other stages according to the AJCC 8th edition criteria (13, 14). A recent study indicated that adjuvant chemotherapy improves the prognosis of patients who have been diagnosed with stage I or II pancreatic cancer. However, its effectiveness is restricted in patients with stage III pancreatic cancer [13]. Nevertheless, another study indicated that postoperative chemotherapy did not confer any benefits to patients with early-stage pancreatic ductal adenocarcinoma (15). Consequently, a substantial number of pancreatic cancer patients undergo surgical resection but do not receive adjuvant chemotherapy. This decision can be influenced by numerous factors. One crucial factor is the unclear efficacy of adjuvant chemotherapy across different stages of the disease. The main aim of this study was to assess the survival advantages that adjuvant chemotherapy provides to patients with pancreatic cancer of different disease stages.

## 2 Methods

### 2.1 Patients

**Figure 1:**
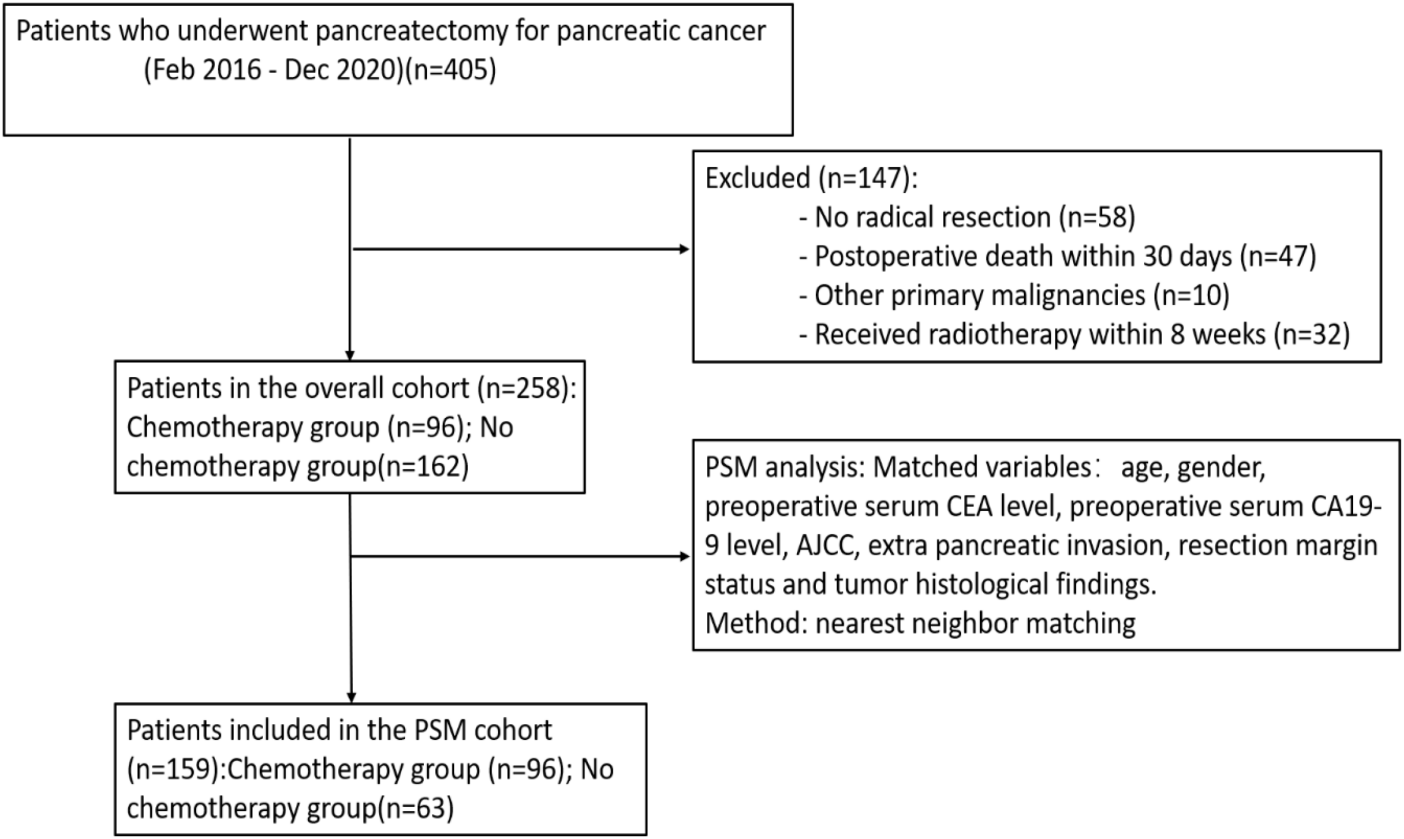
The flow chart of patients with pancreatic cancer enrolled in this study.

We retrospectively reviewed 405 patients who underwent pancreatectomy for pancreatic cancer at the First Affiliated Hospital of Xi’an Jiaotong University between February 2016 and December 2020 and were followed for at least 3 years. Follow-up was conducted every 3 months for the first 2 years post-surgery and every 6 months thereafter, until July 27, 2024. Each visit included clinical examination, serum tumor markers (CEA, CA19-9), and imaging (contrast-enhanced CT or MRI of the chest, abdomen, and pelvis), which was performed semiannually for 2 years and annually thereafter, or more frequently if clinically indicated. Follow-up was conducted through outpatient clinic visits or telephone interviews. Overall survival (OS) was defined as the period from the date of pancreatic cancer diagnosis to the date of death or the time of last follow-up. Patients lost to follow-up were censored at the time of last contact. A total of 105 patients who did not receive pancreatic surgery or died within 30 days after surgery were excluded. Among these patients, 10 patients who had other tumors and 32 patients who received radiotherapy within 8 weeks after surgery were excluded. Finally, 258 patients with resected pancreatic cancer were included in this retrospective study. Patients who received neoadjuvant chemotherapy (n=0) were excluded (**Figure 1**).

Resection margin status was defined according to the standardized protocol of the International Study Group of Pancreatic Surgery (ISGPS): R0 was defined as no tumor cells within 1 mm of the resection margin, R1 as tumor cells within 1 mm of the margin, and R2 as macroscopic residual tumor (16). All 258 included patients had R0 (n=257) or R1 (n=1) resection; patients with R2 resection (n=0) were excluded from the study. A total of 96 patients received postoperative chemotherapy, and 162 patients received conservative treatment without chemotherapy. In addition, the early stage of pancreatic cancer was defined as stage I, and late-stage PDAC were defined as stage II-IV according to the AJCC 8th edition criteria. In our study, 59 patients were diagnosed with stage I pancreatic cancer, and 199 patients were diagnosed with late-stage PDAC. Among the 199 patients with late-stage disease, 10 (5.0%) had distant metastases (M1) at the time of surgery. These patients underwent curative-intent pancreatectomy due to resectable oligometastatic disease (solitary liver metastasis in 8, solitary lung metastasis in 2) and good performance status (ECOG 0-1), in accordance with NCCN guidelines for selected patients with oligometastatic disease. Patients with resected pancreatic cancer were diagnosed on the basis of pathological characteristics and important imaging methods according to the NCCN guidelines (4). Demographic data, including age, sex, AJCC stage, pathological type, tumor size, extrapancreatic invasion (defined pathologically as tumor extension beyond the pancreatic capsule into peripancreatic soft tissue, adjacent organs, or major vessels), resection margin status, nodal status, preoperative serum CEA levels, preoperative serum CA 19–9 levels, operative approach and postoperative lengths of stay, were examined. Adjuvant chemotherapy was defined as chemotherapy that was started within 8 weeks after surgery in patients with a status of R0 or R1 (residual tumor classification).

Patients in the chemotherapy group were treated with AG (gemcitabine 1.4 g, iv, d1, or d8 combined with albumin-paclitaxel 100mg, d1, q3w) or GS (gemcitabine 1.4 g, d1, or d8 combined with tegafur 50 mg po bid d1-14), which was repeated for four cycles at 21-day intervals. Patients in the control group received only radical surgery. Among the 96 patients who received adjuvant chemotherapy, 78 received the AG regimen and 18 received the GS regimen. The median number of chemotherapy cycles completed was 4 (range: 1-6) in the AG group and 4 (range: 1-6) in the GS group. The choice between AG and GS regimens was determined by the treating physicians based on patients’ performance status, comorbidities, and expected tolerability, as this was a retrospective observational study rather than a randomized trial. Both regimens are standard options recommended by NCCN guidelines for adjuvant treatment of pancreatic cancer. The eligibility criteria for adjuvant chemotherapy were as follows: (a) performance status, 1 or 2; (b) adequate bone marrow function (absolute neutrophil count >1500/mm^3^; hemoglobin >9.0 g/dL; and platelets >100,000/mm^3^); (c) adequate hepatic function (bilirubin<2.0 mg/dL; aspartate aminotransferase and alanine aminotransferase ≤100 IU/L); and (d) consent to receive adjuvant chemotherapy. The study was approved by the Ethics Committee of the First Affiliated Hospital of Xi’an Jiao Tong University.

### 2.2 Data analysis and statistics

Numeric data are displayed as medians and ranges. Numbers and percentages are used to describe categorical data. Furthermore, the baseline variables included continuous variables that were analyzed by the paired t test or Mann–Whitney U test and categorical variables that were analyzed by the chi-square test. The influence of postoperative chemotherapy on the prognosis of patients with different stages of pancreatic cancer was analyzed via Kaplan–Meier methods, and the significance was analyzed by the log-rank test. A Cox proportional hazards model of OS was used to estimate the hazard ratio (HR) of postoperative chemotherapy for patients with resectable pancreatic cancer. The 95% confidence intervals (CIs) are also shown.

We used propensity score matching (PSM) with age, gender, preoperative serum CEA level, preoperative serum CA19–9 level, AJCC stage, extrapancreatic invasion, resection margin status and tumor histological findings for patients to balance the baseline characteristics. There were no missing data for any of the matching variables included in the propensity score model. Patients in the chemotherapy group were matched 1:1 to those in the non-chemotherapy group using nearest-neighbor matching without replacement, with a caliper width of 0.2 times the standard deviation of the logit of the propensity score. PSM was performed using SPSS version 29.0 (SPSS, Chicago, IL, USA), and detailed matching procedures are available from the corresponding author upon request. PSM was performed using R software (version 4.2.1; R Foundation for Statistical Computing, Vienna, Austria) with the MatchIt package (version 4.5.0). The R code used for matching is available from the corresponding author upon reasonable request. Cox regression model was established with these variables, including age, chemotherapy, preoperative serum CA19–9 level, extrapancreatic invasion status and resection status, to determine the risk factors for survival among patients with resected pancreatic cancer. A P value less than 0.05 was considered to indicate a significant difference. The statistical analyses were performed in SPSS ver. 29.0 (SPSS, Chicago, IL, USA).

## 3 Results

A total of 258 patients were included in the final analysis. Among these patients, 96 (37%) were administered chemotherapy after pancreatectomy, and 162 (63%) did not receive adjuvant chemotherapy after radical surgery.

### 3.1 Baseline patient information

The patient characteristics are shown in **Table 1**. The mean age of the patients was 61.5 years (range: 26–87 years). The demographic and disease characteristics of these patients treated with and without chemotherapy after pancreatectomy were similar, except that the patients in the chemotherapy group were older (58.8 *vs.* 62.8 years, P = 0.00). Based on the 8th edition of the AJCC, 59 patients (23%) were diagnosed with stage I pancreatic cancer, and 199 patients (77%) were diagnosed with disease of other stages. PSM was performed according to age, gender, preoperative serum CEA level, preoperative serum CA19–9 level, AJCC, extrapancreatic invasion, resection margin status and tumor histological findings. There were no significant differences in the important features (**Table 1**). As shown in **Supplementary Table S1**, most covariates achieved good balance with SMD < 0.1 after matching. However, extrapancreatic invasion (SMD = 0.27) and tumor histological findings (SMD = 0.32) showed residual imbalance. A total of 159 patients were included after PSM. Among these patients, 96 (60%) received chemotherapy after pancreatectomy, and 63 (40%) did not. Thirty-six patients (23%) were diagnosed with stage I pancreatic cancer, and 123 patients (77%) were diagnosed with advanced-stage disease.

**Table 1:** Basic demographic and clinical data of patients with resectable pancreatic cancer who underwent postoperative treatments.

Characteristic	Postoperative therapy before matching (N = 258) No (% row)		P value	Postoperative therapy after matching (N = 159) No (% row)		P value
	Yes (N=96)	No (N=162)		Yes (N=96)	No (N=63)	
Age, yr	58.8	62.8	0.00	58.8	60.5	0.28
Male, Sex	46	95	0.09	46	31	0.87
ECOG performance status			0.55			0.16
0	84	134		84	54	
1	11	26		11	8	
2	1	1		1	1	
Postoperative complications			0.32			1.13
Postoperative hemorrhage	10	12		10	9	
Delayed gastric emptying	6	8		6	5	
Pancreatic fistula	13	16		13	8	
pulmonary infection	1	1		1	0	
Preoperative Serum CEA level (U/mL)	10.15	5.55	0.06	10.15	5.81	0.32
Preoperative serum CA19–9 level (U/mL)	679.19	698.62	0.46	679.19	773.46	0.67
AJCC			0.93			0.59
1	24	35		24	12	
2	67	118		67	49	
3	4	7		4	1	
4	1	2		1	1	
Pathological tumor size (pT)			0.55			0.11
pT1	8	9		8	5	
pT2	31	43		31	10	
pT3	44	86		44	35	
pT4	13	24		13	13	
Nodal status			0.45			0.33
N0	62	116		62	47	
N1	30	39		30	15	
N2	4	7		4	1	
Extrapancreatic invasion	16	7	0.00	16	5	0.11
Resection margin status			0.79			1.00
R0	95	162		95	63	
R1	1	0		1	0	
Tumor histological findings			0.20			0.06
Ductal adenocarcinoma	96	157		96	60	
Nonductal carcinoma	0	5		0	3	
Type of pancreatectomy			0.98			0.96
Partial	6	10		6	3	
Total	90	152		90	60	
Postoperative Length of stay	22	24	0.06	22	22	0.53
Survival			0.18			0.02
yes	32	38		32	10	
no	64	123		64	53	
nr	0	1		0	0	
Median survival, month (range)	17.20 (10.66-23.74)	13.03 (11.07-15.00)	0.02	17.20 (10.66-23.74)	11.93 (8.82-15.05)	0.01

### 3.2 Overall survival of total patients and in different treatment groups

**Figure 2:**
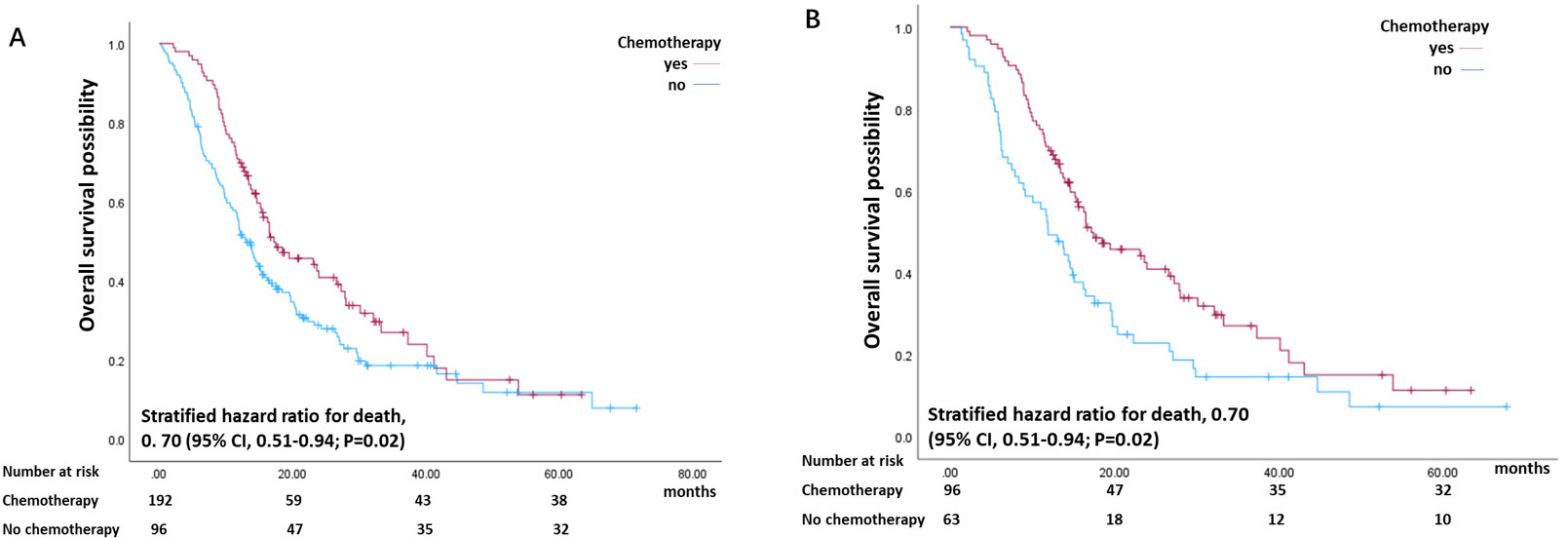
Kaplan–Meier estimate of overall survival stratified by chemotherapy status (yes or no) before PSM **(A)** and after PSM **(B)**.

Among the 258 patients, 187 patients died during follow-up (**Table 1**). The median survival of the patients we included was 14.90 (range, 13.16–16.64) months (**Table 1**). Furthermore, the median survival time was 17.20 (range, 10.66–23.74) months for patients who received chemotherapy and 13.03 (range, 11.07–15.00) months for patients who did not receive chemotherapy (P = 0.03) (**Table 1**). In addition, as shown in **Figure 2A**, the overall survival of the chemotherapy group was greater than that of the control group (stratified hazard ratio for death, 0.70 (95% CI, 0.51–0.94; P = 0.02)). The cumulative OS rates at 1, 3 and 5 years in the chemotherapy and control groups were 70% and 53%, 24% and 16%, and 11% and 0.8%, respectively (**Figure 2A**). In the unmatched cohort of all evaluated patients, age and preoperative serum CA199 level were correlated with OS in the Cox proportional hazards model (P = 0.01) (**Table 2**).

**Table 2:** Multivariate cox proportional hazards regression of overall survival before PSM.

Characteristic	Multivariate		
	Hazard ratio	95% CI for HR	P value
Age, yr	1.03	1.01-1.04	0.01
Chemotherapy (yes)	0.74	0.52-1.04	0.08
Preoperative serum CA199 level, U/mL	1.00	1.00-1.00	0.01
Extrapancreatic invasion (yes)	0.62	0.30-1.27	1.27
Resection status (R1)	0.00	0.00-6.73	0.96

After PSM, 159 patients were included, and 117 patients died during follow-up (**Table 1**). The median survival of the patients was 15.33 (range, 13.43–17.23) months (**Table 1**). Furthermore, patients who received adjuvant chemotherapy had longer median survival than patients who did not receive chemotherapy after surgery (P = 0.01) (**Table 1**). In addition, the overall survival of the chemotherapy group was greater than that of the control group (stratified hazard ratio for death, 0.70 (95% CI, 0.51–0.94; P = 0.02)) (**Figure 2B**). The cumulative OS rates at 1, 3 and 5 years in the chemotherapy and control groups were 69% and 49%, 27% and 14%, and 11% and 7%, respectively (**Figure 2B**). According to the Cox proportional hazards model, age was a poor prognostic factor for OS (P = 0.01). However, there was an obvious correlation between postoperative chemotherapy and the overall survival of patients with resected pancreatic cancer (hazard ratio (HR) 0.62, 95% CI 0.42–0.91; P = 0.01) (**Table 3**).

**Table 3:** Multivariate cox proportional hazards regression of overall survival after PSM.

Characteristic	Multivariate		
	Hazard ratio	95% CI for HR	P value
Age, yr	1.02	1.00-1.04	0.03
Chemotherapy (yes)	0.62	0.42-0.91	0.01
Preoperative serum CA199 level, U/mL	1.00	1.00-1.00	0.05
Extrapancreatic invasion (yes)	0.56	0.26-1.23	0.15
Resection status (R1)	0.00	0.00-1.40	0.96

### 3.3 Overall survival of patients with stage I disease and in different treatment groups

**Figure 3:**
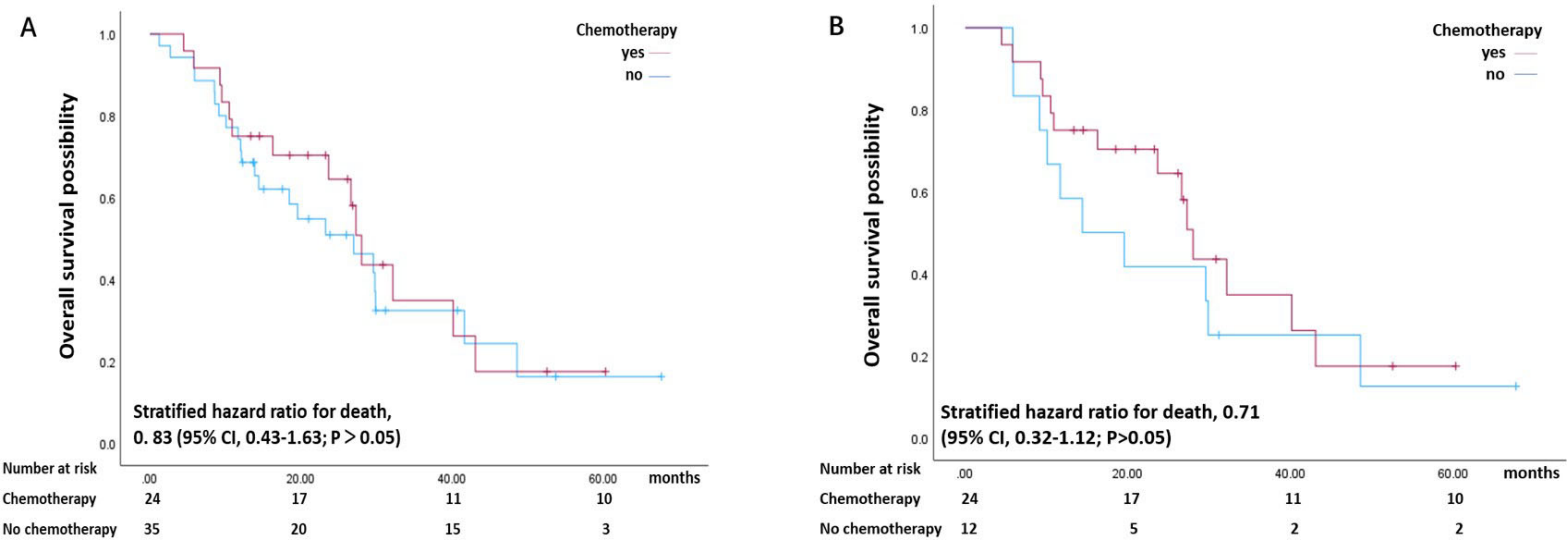
Kaplan–Meier estimate of overall survival of patients with early stage pancreatic cancer stratified by chemotherapy status (yes or no) before PSM **(A)** and after PSM **(B)**.

For patients with stage I disease, 59 patients were included, and 36 patients died during this study. The median survival of the patients with early stage disease that were analyzed was 27.27 (range, 21.20–33.33) months (**Table 1**). Furthermore, the median survival was 28.00 months for patients who received chemotherapy and 26.97 months for patients who did not receive chemotherapy (P>0.05) (**Figure 3A**). In addition, for patients with stage I disease, the overall survival rate of the chemotherapy group was not significantly different from that of the group that did not receive chemotherapy (stratified hazard ratio for death, 0. 83 (95% CI, 0.43–1.63; (P>0.05)). The cumulative OS rates at 1, 3 and 5 years in the chemotherapy and control groups were 75% and 69%, 26% and 32%, and 17% and 16%, respectively (P>0.05) (**Figure 3A**). Similarly, adjuvant chemotherapy was not related to the OS of patients with early-stage disease according to the multivariate analyses (P>0.05) (**Table 4**).

**Table 4:** Multivariate cox proportional hazards regression of overall survival of patients with early stage pancreatic cancer before PSM.

Characteristic	Multivariate		
	Hazard ratio	95% CI for HR	P value
Age, yr	1.25	1.00-1.04	0.35
Chemotherapy (yes)	0.69	0.27-1.77	0.44
Preoperative serum CA199 level, U/mL	1.00	1.00-1.00	0.41
Extrapancreatic invasion (yes)	1.25	0.15-10.18	0.83

After PSM, 36 patients who underwent pancreatic resection at very early stages of pancreatic cancer were included. Among these patients, 24 patients died during follow-up (**Figure 3B**). The median survival of the patients was 27.26 (range, 20.83–33.70) months (**Figure 3B**). In addition, the median survival did not significantly differ between the group treated with chemotherapy and the group not treated with chemotherapy (P>0.05) (**Table 5**). The overall survival rates of the patients treated with or without chemotherapy coincided, as shown in **Figure 3B** (HR 0.71; 95% CI 0.32–1.12; P = 0.41)). The cumulative OS rates at 1, 3 and 5 years in the chemotherapy and control groups were 75% and 71%, 35% and 24%, 17% and 16%, respectively (**Figure 3B**). According to the Cox proportional hazards model, there was no correlation between postoperative chemotherapy and the overall survival of patients with resected stage I pancreatic cancer (hazard ratio (HR) 0.69 95% CI 0.27–1.77; P>0.05) (**Table 5**).

**Table 5:** Multivariate cox proportional hazards regression of overall survival of patients with early stage pancreatic cancer after PSM.

Characteristic	Multivariate		
	Hazard ratio	95% CI for HR	P value
Age, yr	1.02	0.97-1.07	0.35
Chemotherapy (yes)	0.69	0.27-1.77	0.45
Preoperative serum CA199 level, U/mL	1.00	1.00-1.00	0.41
Extrapancreatic invasion (yes)	1.25	0.15-10.18	0.83

### 3.4 Overall survival of patients with late-stage disease and in different treatment groups

**Figure 4:**
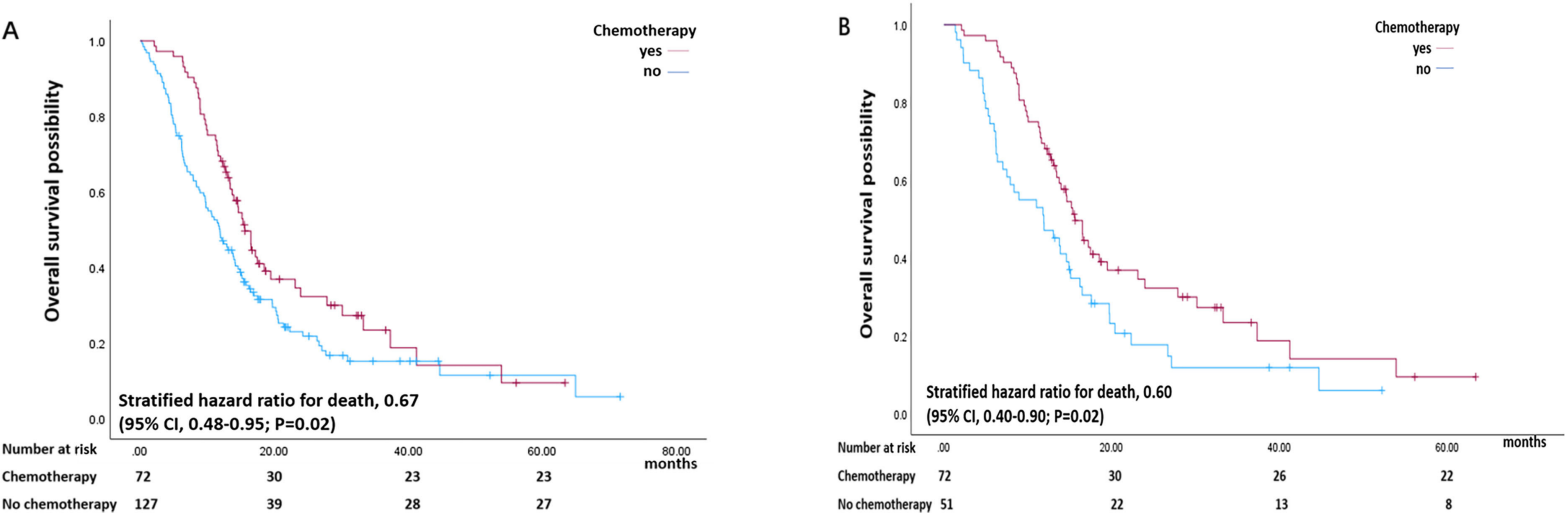
Kaplan–Meier estimate of overall survival of patients with late-stage pancreatic cancer stratified by chemotherapy status (yes or no) before PSM **(A)** and after PSM **(B)**.

A total of 199 patients with late-stage PDAC (stages II-IV according to the AJCC 8th edition) were included. The median survival of these patients was 13.73 months (**Table 6**). Furthermore, the median survival was 15.60 months for patients who received chemotherapy, whereas it was 11.90 months for patients who did not receive chemotherapy (P<0.05) (**Figure 4A**). In addition, for late-stage patients, the overall survival of the chemotherapy group was significantly different from that of the group that was not treated with chemotherapy (stratified hazard ratio for death, 0.67 (95% CI, 0.48–0.95; P<0.05) (**Figure 4A**). The cumulative OS rates at 1, 3 and 5 years in the chemotherapy and control groups were 68% and 47%, 23% and 22%, and 0.9% and 0.5%, respectively (P<0.05) (**Figure 4A**). Furthermore, multivariate analyses suggested that adjuvant chemotherapy was an independent predictor of OS (P<0.05) (**Table 6**).

**Table 6:** Multivariate cox proportional hazards regression of overall survival of patients with late-stage pancreatic cancer before PSM.

Characteristic	Multivariate		
	Hazard ratio	95% CI for HR	P value
Age, yr	1.02	1.00-1.05	0.68
Chemotherapy (yes)	0.61	0.40-0.95	0.03
Preoperative serum CA199 level, U/mL	1.00	1.00-1.00	0.05
Extrapancreatic invasion (yes)	0.46	0.20-1.05	0.07
Resection status (R1)	0.00	0.00-5.62	0.97

In the chemotherapy group versus the control group after PSM, 72 patients with late-stage disease received chemotherapy, and 51 patients with advanced-stage disease did not receive chemotherapy. Patients who received chemotherapy had significantly better survival than those who did not (HR 0.60; 95% CI 0.40–0.90; P = 0.02) (**Figure 4B**). The cumulative OS rates at 1, 3 and 5 years in the chemotherapy and control groups were 68% and 48%, 23% and 15%, and 0.9% and 0.6%, respectively (P<0.05) (**Figure 4B**). According to the Cox proportional hazards model, there was a correlation between postoperative chemotherapy and the overall survival of patients with resected pancreatic cancer of an advanced stage (hazard ratio (HR) 0.59 95% CI 0.38–0.91; P = 0.02) (**Table 7**).

**Table 7:** Multivariate cox proportional hazards regression of overall survival of patients with late-stage pancreatic cancer after PSM.

Characteristic	Multivariate		
	Hazard ratio	95% CI for HR	P value
Age, yr	1.02	0.20-1.07	0.08
Chemotherapy (yes)	0.59	0.38-0.91	0.02
Preoperative serum CA199 level, U/mL	1.00	1.00-1.00	0.13
Extrapancreatic invasion (yes)	0.46	0.20-1.07	0.07

## 4 Discussion

Pancreatic cancer continues to be a highly lethal malignancy primarily because it is often diagnosed at a late stage and has a strong tendency to recur and metastasize. Surgical treatment is considered the first-line approach for curing pancreatic cancer (17). However, the significance of adjuvant chemotherapy must not be overlooked because the overall survival (OS) of patients who have undergone radical pancreatic resection remains poor (18). Among the various postoperative treatment options, adjuvant chemotherapy has been associated with improved survival of pancreatic cancer patients, as demonstrated by the CONKO-001 trial (18). Our data indicate that pancreatic cancer patients can benefit from postoperative chemotherapy, as this treatment increases their OS. This improvement persists even after conducting a Cox proportional hazards regression analysis and when the analysis is performed on the matched data.

Importantly, limited emerging evidence supports the administration of postoperative chemotherapy to patients with resected and borderline resected pancreatic cancer, particularly in those with stage I pancreatic cancer (19). In contrast to the ESPAC trials, in which only 10% (104/1088) of included patients had stage I pancreatic cancer (20), our study included 23% (59/258) of patients with stage I pancreatic cancer. Few studies have identified an overall survival benefit of adjuvant chemotherapy in patients with stage 1A pancreatic ductal adenocarcinoma (PDAC) (5). Although a six-month adjuvant chemotherapy regimen based on gemcitabine is recommended for patients who can tolerate it, most patients are unable to complete the full course of chemotherapy. For example, in the CONKO-001 trial, at least 16% of patients did not complete postoperative chemotherapy due to postoperative complications and adverse events, which were associated with age and the burden of comorbidities (8). Additionally, a randomized trial revealed that 10% of patients with post-surgery complications did not receive adjuvant therapy (21). In our study, only 96 of the 258 patients received postoperative chemotherapy. Moreover, postoperative chemotherapy with modified FOLFIRINOX or the gemcitabine regimen is associated with excessive toxicity, which may offset the potential benefits of chemotherapy (20). Additionally, severe adverse events such as marrow suppression and digestive tract reactions have occurred in patients who receive fluorouracil plus folinic acid (22).

Our study revealed that patients with very early-stage pancreatic cancer may not benefit from postoperative chemotherapy, which is consistent with a recent Frontiers study by Zhang et al. showing that stage IA patients did not benefit from chemotherapy (15). The observed disparity in chemosensitivity between early-stage and late-stage tumors can be attributed to several interrelated biological factors. Advanced malignancies often exhibit a higher proliferative index, rendering them more susceptible to chemotherapeutic agents. Additionally, patients with advanced disease harbor a greater burden of micrometastases (23). Furthermore, advanced cancers frequently develop a dense desmoplastic stroma, a structure that can be effectively targeted by agents such as paclitaxel, thereby enhancing drug penetration. Studies have also demonstrated that during pancreatic cancer progression, widespread epigenetic reprogramming occurs in parallel with subclonal evolution, leading to heterogeneous metabolic, epigenetic, and malignant properties (24). Finally, treatment tolerability remains a critical consideration, as older patients with early-stage disease may be more vulnerable to toxicity, potentially compromising treatment completion and reducing efficacy. Thus, on one hand, given the limited efficacy of postoperative chemotherapy observed in our cohort of patients with early stage pancreatic cancer, our data suggest that adjuvant chemotherapy may not confer a significant survival benefit in this subgroup. On the other hand, for patients with other stages of pancreatic cancer, surgical management should aim to minimize comorbidities, and a chemotherapy regimen should be designed to reduce toxic side effects to the greatest extent possible.

To a large extent, patients frequently have advanced disease at the time of diagnosis because pancreatic cancer cells tend to metastasize, and this type of micrometastasis is difficult to detect through clinical imaging. Additionally, patients in the early stages generally do not exhibit obvious symptoms (25). Despite the fact that pancreatic ductal adenocarcinoma is a lethal disease with a rising incidence rate, population-based screening is not advocated (26). Nevertheless, it is significant to selectively monitor high-risk individuals. These include those with a family history of PDAC or those with a genetic predisposition resulting from inherited pathogenic variants such as germline CDKN2A, P53, and SMAD4 variants (27–29). Although our study did not investigate neoadjuvant therapy, it is worth noting that the role of neoadjuvant approaches in pancreatic cancer is evolving. We hypothesized that if preoperative treatment can downgrade the stage of pancreatic cancer to stage I, adjuvant chemotherapy may not be essential after surgery. Upfront surgery combined with adjuvant therapy may offer certain advantages. First, neoadjuvant treatment could increase the likelihood of achieving microscopically complete (R0) resection. Second, neoadjuvant therapy might prevent unnecessary surgery in patients with rapidly progressive disease. However, this remains a hypothesis that requires testing in prospective clinical trials and is not supported by data from the present study. Current evidence and guidelines continue to support adjuvant chemotherapy for all resected pancreatic cancer patients, regardless of stage, until high-quality data suggest otherwise.

Our study had limitations. First, this was a retrospective study with a relatively small sample size, and the impact of postoperative complications on OS was not assessed. Second, we did not account for the potential influence of chemotherapeutic agents or the number of chemotherapy cycles. Third, - Although we employed propensity score matching to minimize confounding, this study remains subject to potential unmeasured confounding and indication bias. PSM can only account for observed covariates included in the propensity score model. Factors such as detailed performance status, frailty, nutritional status, social support, and physician judgment—which may have influenced both the decision to administer chemotherapy and patient outcomes—were not fully captured in our analysis. Moreover, the inherent selection bias in favor of healthier patients receiving chemotherapy may have overestimated the true treatment effect, even after matching. These findings require validation in larger, multicenter cohorts and, ideally, prospective randomized controlled trials. Lastly, although we employed propensity score matching to minimize confounding, some degree of residual imbalance remained for certain covariates. After matching, extrapancreatic invasion showed a standardized mean difference (SMD) of 0.27, and tumor histological findings showed an SMD of 0.32 (**Supplementary Table S1**). Although SMD values < 0.1 are generally considered to indicate good balance, values between 0.1 and 0.2 may be acceptable, and values > 0.2 suggest meaningful residual imbalance. Specifically, the imbalance in extrapancreatic invasion—a known poor prognostic factor—may have biased the comparison between groups. Patients in the chemotherapy group had a higher proportion of extrapancreatic invasion after matching (58/96, 60.4%) compared with the no-chemotherapy group (31/63, 49.2%), which would tend to underestimate the true benefit of chemotherapy. Conversely, the imbalance in histological findings, with all chemotherapy patients having ductal adenocarcinoma compared with 95% in the no-chemotherapy group, may have introduced bias in the opposite direction.

## 5 Conclusions

In conclusion, in patients with resected pancreatic cancer, chemotherapy is beneficial for patients with late-stage pancreatic cancer, but these benefits are limited the early-stage pancreatic cancer.

## Data Availability

The raw data supporting the conclusions of this article will be made available by the authors, without undue reservation.
