# Supplementary material for: Post-pancreatectomy chemotherapy improves the survival of patients with late-stage but not early-stage pancreatic cancer
Source: Front Oncol. 2026 Apr 13;16:1744027. doi: 10.3389/fonc.2026.1744027 (PMC13111022; doi:10.3389/fonc.2026.1744027)
Supplement: Supplementary file 1 [file Table1.pdf]

Supplementary Material

Table S1. Standardized mean differences of covariates before and after propensity score matching

Characteristic	Before PSM			After PSM		
	Chemo (n=96)	No Chemo (n=162)	SMD	Chemo (n=96)	No Chemo (n=63)	SMD
Age, mean	58.8	62.8	0.52	58.8	60.5	0.1
Male	46	95	0.22	46	31	0.02
Preoperative CEA, mean (U/mL)	10.15	5.55	0.18	10.15	5.81	0.1
Preoperative CA19-9, mean (U/mL)	679.19	698.62	0.06	679.19	773.46	0.1
AJCC stage			0.08			0.08
Stage I	24	35		24	12	
Stage II	67	118		67	49	
Stage III	4	7		4	1	
Stage IV	1	2		1	1	
Extrapancreatic invasion	16	7	0.41	16	5	0.27
R0 resection	95	162	0.14	95	63	0.08
Tumor histological findings			0.25			0.32
Ductal adenocarcinoma	96	157		96	60	
Nonductal carcinoma	0	5		0	3	
